# Emergence of a piliated and multidrug-resistant *Streptococcus pneumoniae* serotype 35B-ST156 clone in Japan

**DOI:** 10.1128/spectrum.00632-25

**Published:** 2025-08-05

**Authors:** Haruko Miyazaki, Satoshi Nakano, Rie Shibuya, Bin Chang, Yoshitsugu Miyazaki, Tetsuya Matsumoto, Yukihiro Akeda

**Affiliations:** 1 Department of Microbiology, Tokyo Medical University, Tokyo, Japan; 2 Antimicrobial Resistance Research Center Laboratory 7, National Institute of Infectious Diseases, Tokyo, Japan; 3 Department of Clinical Laboratory, Saiseikai Yokohamashi Tobu Hospital, Kanagawa, Japan; 4 Department of Bacteriology 1, National Institute of Infectious Diseases, Tokyo, Japan; 5 Pathogen Genomics Center, National Institute of Infectious Diseases, Tokyo, Japan; 6 Department of Infectious Diseases, International University of Health and Welfare, Chiba, Japan; The University of Texas at Tyler, Tyler, Texas, USA

**Keywords:** *Streptococcus pneumoniae*, bacterial typing, whole-genome sequence, serotype 35B-ST156, antimicrobial resistance, vaccine

## Abstract

*Streptococcus pneumoniae* serotype 35B, a nonvaccine serotype, is common among clinical pneumococcal isolates in Japan after vaccination. We report the emergence of serotype 35B sequence type 156 (35B-ST156), a rare type previously reported in Japan, in isolates from patients at a hospital. Multilocus sequence typing of serotype 35B isolates from 2014 to 2023 revealed that 35B-ST156 appeared in 2019 and was the most common sequence type of 35B in 2023. All 17 isolates of 35B-ST156 had the type 1 pilus, an adhesion factor, and were nonsusceptible to β-lactams, erythromycin, and co-trimoxazole. Whole-genome sequencing analysis demonstrated that the isolates harbored type 1 pilus and exhibited resistance profiles for β-lactam (*pbp1a*: 4, *pbp2b*: 12, and *pbp2x*: 7), macrolide (*mefE*), and co-trimoxazole (*folA* mutation and *folP* insertion). The analysis, along with global ST156 isolates in the database, suggested that the 35B-ST156 isolates in the present study were closely related to the strains prevalent in the United States, which had been generated by capsular switching, and clonally spread in the region through repeated small mutations. An increase in the proportion of 35B-ST156 influenced the antimicrobial susceptibility pattern of serotype 35B. The increased prevalence of ST156 in nonvaccine serotypes may alter antimicrobial efficacy against pneumococcal infections in Japan. Therefore, this clone should be monitored for effective treatment and prevention of pneumococcal infections.

**IMPORTANCE:**

Although an increase in the prevalence of *Streptococcus pneumoniae* 35B-ST156 has not been reported in Japan, we found that ST156 was the most common sequence type of 35B among clinical isolates at a hospital in 2023. All 35B-ST156 isolates had a type 1 pilus and were multidrug resistant. Whole-genome sequencing analysis of the 35B-ST156 isolates showed that these isolates were closely related to the strains in the United States, generated by capsular switching and prevalent after 13-valent pneumococcal conjugate vaccine implementation, and clonally spread in the region. Furthermore, an increase in the proportion of 35B-ST156 influenced the antimicrobial susceptibility pattern of serotype 35B. The results provide useful information for guiding the treatment and prevention of pneumococcal infections.

## INTRODUCTION

*Streptococcus pneumoniae* is a commensal bacterium of the human upper respiratory tract and is the causative pathogen of meningitis, pneumonia, bacteremia, and otitis media. Invasive pneumococcal disease (IPD) (e.g., meningitis and bacteremia) is a rapidly progressive disease with a high mortality (1, 2). Pneumococcal vaccines are highly effective against pneumococcal diseases; however, the increasing numbers of nonvaccine-type infections after vaccine introduction are a concern (3–5). In Japan, vaccination with the 13-valent pneumococcal conjugate vaccine (PCV13) and the 23-valent pneumococcal polysaccharide vaccine became routine for children in 2013 and for older adults in 2014, respectively, according to the national vaccine program. *S. pneumoniae* serotype 35B, a nonvaccine serotype, has become one of the most prevalent serotypes among clinical isolates from pneumococcal infections in Japan following vaccination implementation (6). Previously, we reported that serotype 35B was the most common serotype in post-vaccination clinical isolates at a hospital, with 88.9% being penicillin nonsusceptible (7). We also previously reported that during 2014-2018, the major sequence type (ST) of the 35B serotypes was ST558, which was both piliated and multidrug resistant (8). In our 10-year (2014–2023) investigation of the serotype distribution of pneumococcal isolates from individuals and analysis of the ST of serotype 35B, we have observed the appearance and increase in the proportion of ST156 in serotype 35B isolates. Between 2019 and 2022, we identified four serotype 35B ST156 (35B-ST156) isolates that, like ST558, carried the type 1 pilus gene and exhibited higher minimum inhibitory concentrations (MICs) for β-lactams than ST558 strains (9). In 2023, ST156 became the most common ST among our serotype 35B isolates. Although an increase in the prevalence of 35B-ST156 isolates has been reported in the United States after PCV13 implementation (10), an increase in 35B-ST156 prevalence in Japan has not been reported. Therefore, we performed whole-genome sequencing analysis of the 35B-ST156 isolates to clarify their details. In addition, the effect of an increased distribution of 35B-ST156 on the characteristics of serotype 35B was examined.

## MATERIALS AND METHODS

### Isolates and serotyping

From 2014 to 2023, 1,344 *S*. *pneumoniae* isolates were collected from clinical specimens at a sentinel hospital in Kanagawa Prefecture, Japan, regardless of patient age or diagnosis. If multiple isolates were obtained from the same patient within 3 months, the strain from a sterile specimen or the first strain was selected. A total of 1,261 strains were used in this study, of which 95.4% were derived from sputum, bronchial, or nasopharyngeal samples; 3.7% were derived from blood, cerebrospinal fluid, or pleural effusion of patients with IPD; and 0.9% were obtained from otorrhea or others. Isolates were cultured on 5% blood agar plates at 37°C in a 5% CO_2_ environment. DNA was extracted using the Cica Geneus DNA Extraction Reagent (Kanto Chemical Co., Tokyo, Japan) according to the manufacturer’s instructions. Capsular serotypes were identified via sequential multiplex PCR (11) using a QIAGEN Multiplex PCR Kit (QIAGEN, Hilden, Germany) or via the Quellung reaction with serotype-specific rabbit antisera (Statens Serum Institut, Copenhagen, Denmark).

### Multilocus sequence typing and pilus assay

The STs of *S. pneumoniae* isolates were determined based on the sequences of seven housekeeping genes (*aroE, gdh*, *gki*, *recP*, *spi*, *xpt*, and *ddl*) (12). Allelic numbers and STs were assigned according to the pneumococcal multilocus sequence typing (MLST) website (https://pubmlst.org/spneumoniae/). Strains for which ≥5 of the 7 alleles were identical were classified as belonging to a clonal complex (CC).

Type 1 pilus positivity was detected via PCR amplification of the subunit gene *rrgC* using primers designed by Regev-Yochay et al. (13) and Quick Taq HS DyeMix (TOYOBO, Osaka, Japan) (8). Type 2 pilus positivity was detected via PCR amplification of the *sipA* according to Aguiar et al. (14). PCR was performed on a Veriti 96-well thermal cycler (Applied Biosystems, Foster City, CA, USA).

### Whole-genome sequencing

Total genomic DNA was extracted from the isolates using the QIAamp DNA MiniKit (QIAGEN). Multiplexed samples were sequenced on an Illumina NovaSeq X Plus platform for 300 cycles, generating 150 bp paired-end reads. We analyzed these read data using the in-house pneumococcal genome analysis pipeline described previously (15–19). In brief, after quality control using fastp v.0.23.1 (reads with average quality [<Q25] were trimmed) (20), the reads were assembled with standard parameters using shovill 1.0.9 (https://github.com/tseemann/shovill). We performed MLST, resistance gene identification (the presence of *ermB*, *ermTR, mefA*, *mefE*, *tetM*, *tetO*, mutations within the *pbp1a*, *pbp2b*, *pbp2x*, *folA*, *folP*, *gyrA*, and *parC* genes), and pilus detection (*rrgA* for type 1 pilus and *pitB-1* for type 2 pilus), as described previously. In addition, we searched resistance genes comprehensively using the ResFinder database (21) implemented in ABRicate (https://github.com/tseemann/abricate) ( the database was downloaded on 20 October 2024). A recombination site-censored maximum likelihood tree was constructed for the tested isolates along with the global ST156 isolates listed in the Pathogenwatch database (https://pathogen.watch/) (accessed on 6 January 2025) using Gubbins v.3.1.3 with standard parameters. The resulting tree was visualized using Interactive Tree Of Life (22).

### Bioassay for antimicrobial susceptibility testing

Antimicrobial susceptibility testing was performed using the MICroFAST Panel Type7 (Beckman Coulter, Brea, CA, USA). Isolates were considered susceptible, intermediate, or resistant to antimicrobial agents according to the susceptibility breakpoints of Clinical Laboratory and Standards Institute (23) and European Committee on Antimicrobial Susceptibility Testing (EUCAST) (24) for MIC. Isolates with intermediate susceptibility or resistance were considered nonsusceptible.

### Statistical analysis

Statistical analyses were performed using the 2 × 2 chi-square test and Fisher’s exact test. Statistical significance was set at *P* < 0.05.

## RESULTS

### Frequency of serotype 35B isolates and STs

**Fig 1:**
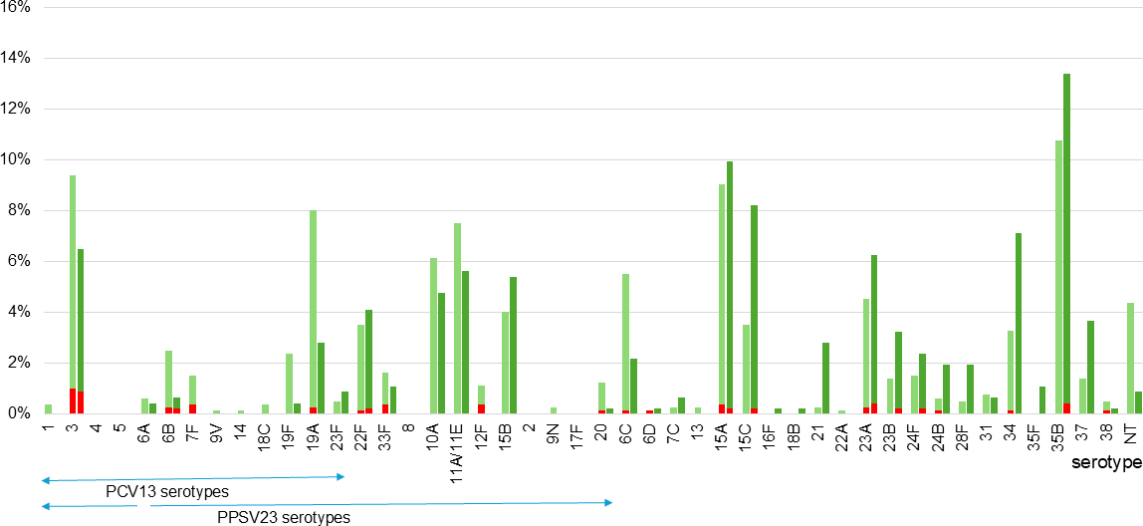
Serotype distribution of pneumococcal strains isolated from 2014 to 2023 (*n* = 1,261). The left and right bars indicate frequencies of serotypes from 2014 to 2018 (*n* = 798) and from 2019 to 2023 (*n* = 463), respectively. The red bars indicate isolates from normally sterile sites. The green bars indicate isolates from nonsterile sites.

**Fig 2:**
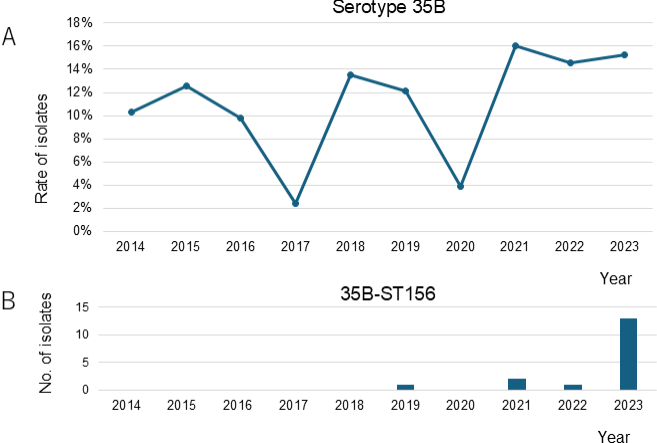
Serotype 35B frequency in pneumococcal isolates and 35B-ST156 isolation between 2014 and 2023. (**A**) Serotype 35B frequency in all isolates by year. (**B**) 35B-ST156 isolates by year.

**Fig 3:**
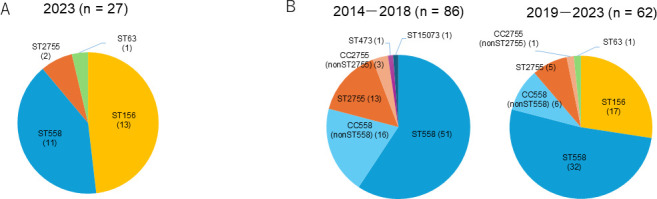
Distributions of sequence type (ST) in serotype 35B isolates. (**A**) Distribution of STs in 2023 isolates. (**B**) Distributions of STs in 2014–2018 and 2019–2023 isolates. CC558, clonal complex 558 (ST558 and STs, for which ≥5 of the seven alleles were identical to ST558); CC2755, clonal complex 2755 (ST2755 and STs, for which ≥5 of the seven alleles were identical to ST2755). Number in brackets: number of strains.

Serotype distributions of pneumococcal isolates from 2014 to 2018 and from 2019 to 2023 are shown in Fig. 1. Serotype 35B accounted for 148 (11.7%) of the total isolates from 2014 to 2023 and was the most frequent serotype. Two 35B isolates (2 out of 148, 1.4%) were from sterile sites (blood from a 95-year-old female in 2019 and blood from a 71-year-old male in 2020) (Fig. 1). The frequency of serotype 35B in all serotypes per year is shown in Fig. 2A. In 2023, serotype 35B accounted for 15.3% (27 out of 177) of the isolates and was the most common serotype. Within our study period, 35B-ST156 (*aroE*: 7, *gdh*: 11, *gki*: 10, *recP*: 1, *spi*: 6, *xpt*: 8, and *ddl*: 1) first appeared in 2019 and significantly increased in frequency by 2023 (Fig. 2B). All 35B-ST156 were isolated from sputum. Figure 3 shows the distribution of STs in serotype 35B. In 2023, MLST revealed that ST156 was the most common ST, accounting for 48.1% (13 out of 27) of the 35B isolates (Fig. 3A). That led to 35B-ST156 accounting for 27.4% of the 35B isolates in 2019–2023 (Fig. 3B). Meanwhile, the percentages of CC558 (ST558 [*aroE*: 18, *gdh*: 12, *gki*: 4, *recP*: 44, *spi*: 14, *xpt*: 77, and *ddl*: 97] and STs, for which ≥5 of the 7 alleles were identical to ST558) and CC2755 (ST2755 [*aroE*: 10, *gdh*: 12, *gki*: 2, *recP*: 1, *spi*: 152, *xpt*: 28, and *ddl*: 14] and STs, for which ≥5 of the 7 alleles were identical to ST2755) in 35B isolates decreased in 2019–2023 compared to 35B isolates in 2014–2018 (*P* = 0.028 and *P* = 0.203, respectively) (Fig. 3B).

### Genomic characteristics of 35B-ST156 isolates

We analyzed the whole-genome sequences of the 17 isolates of 35B-ST156 for subsequent characterization using Pathogenwatch (https://pathogen.watch) and the Centers for Disease Control and Prevention (USA) PBP-type database (https://www.cdc.gov/streplab/pneumococcus/mic.html) (Table 1). These ST156 isolates harbored type 1 pilus and exhibited resistance profiles for β-lactam (*pbp1a*: 4, *pbp2b*: 12, and *pbp2x*: 7), macrolide (*mefE*), and co-trimoxazole (*folA* mutation and *folP* insertion) (Table 1).

**TABLE 1:** Whole-genome sequence analysis of *S. pneumoniae* serotype 35B-ST156 isolates^*a*^

					Allelic profile									PBP profile			Resistance determinant profile									Pili	
Strain no.	Year	Sex	Age	Serotype	*aroE*	*gdh*	*gki*	*recP*	*spi*	*xpt*	*ddl*	ST	GPSC	*1a*	*2b*	*2x*	*ermB*	*ermTR*	*mef*	*tetM*	*tetO*	*folA* mutation	*folP* insertion	*gyrA*	*parC*	Type 1	Type 2
1125	2019	M	78	35B	7	11	10	1	6	8	1	156	6	4	12	7	−	−	E	−	−	+	+	−	−	+	−
1266	2021	F	1	35B	7	11	10	1	6	8	1	156	6	4	12	7	−	−	E	−	−	+	+	−	−	+	−
1273	2021	M	2	35B	7	11	10	1	6	8	1	156	6	4	12	7	−	−	E	−	−	+	+	−	−	+	−
1328	2022	F	0	35B	7	11	10	1	6	8	1	156	6	4	12	7	−	−	E	−	−	+	+	−	−	+	−
1434	2023	M	4	35B	7	11	10	1	6	8	1	156	6	4	12	7	−	−	E	−	−	+	+	−	−	+	−
1442	2023	F	0	35B	7	11	10	1	6	8	1	156	6	4	12	7	−	−	E	−	−	+	+	−	−	+	−
1445	2023	M	3	35B	7	11	10	1	6	8	1	156	6	4	12	7	−	−	E	−	−	+	+	−	−	+	−
1470	2023	M	1	35B	7	11	10	1	6	8	1	156	6	4	12	7	−	−	E	−	−	+	+	−	−	+	−
1498	2023	F	1	35B	7	11	10	1	6	8	1	156	6	4	12	7	−	−	E	−	−	+	+	−	−	+	−
1517	2023	F	1	35B	7	11	10	1	6	8	1	156	6	4	12	7	−	−	E	−	−	+	+	−	−	+	−
1518	2023	M	63	35B	7	11	10	1	6	8	1	156	6	4	12	7	−	−	E	−	−	+	+	−	−	+	−
1526	2023	F	3	35B	7	11	10	1	6	8	1	156	6	4	12	7	−	−	E	−	−	+	+	−	−	+	−
1530	2023	F	1	35B	7	11	10	1	6	8	1	156	6	4	12	7	−	−	E	−	−	+	+	−	−	+	−
1559	2023	M	6	35B	7	11	10	1	6	8	1	156	6	4	12	7	−	−	E	−	−	+	+	−	−	+	−
1572	2023	M	1	35B	7	11	10	1	6	8	1	156	6	4	12	7	−	−	E	−	−	+	+	−	−	+	−
1573	2023	M	2	35B	7	11	10	1	6	8	1	156	6	4	12	7	−	−	E	−	−	+	+	−	−	+	−
1582	2023	M	1	35B	7	11	10	1	6	8	1	156	6	4	12	7	−	−	E	−	−	+	+	−	−	+	−

^*a*^ +, positive for resistance gene or pilus gene; -, negative for resistance gene or pilus gene.

**Fig 4:**
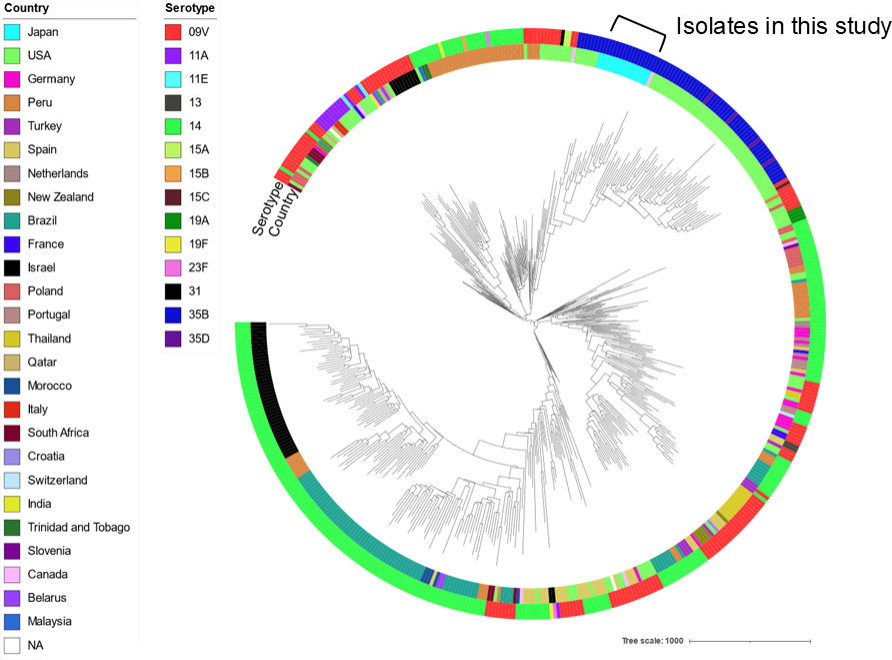
Recombination site-censored maximum likelihood tree for the tested isolates along with global ST156 isolates. We used the global ST156 isolates listed in the Pathogenwatch database and constructed the tree using Gubbins v.3.1.3 with standard parameters. The resulting tree was visualized using Interactive Tree Of Life.

Figure 4 shows the recombination site-censored maximum likelihood tree for the tested isolates, along with the 489 global ST156 isolates. The results suggest that the 35B-ST156 isolates in this study were strains closely related to the 35B-ST156 strains in the United States and Canada caused by capsular switching and that the clone has spread in the region (Fig. 4).

### Clinical information, pilus positivity, and antimicrobial susceptibility of serotype 35B isolates and 35B-ST156

**Fig 5:**
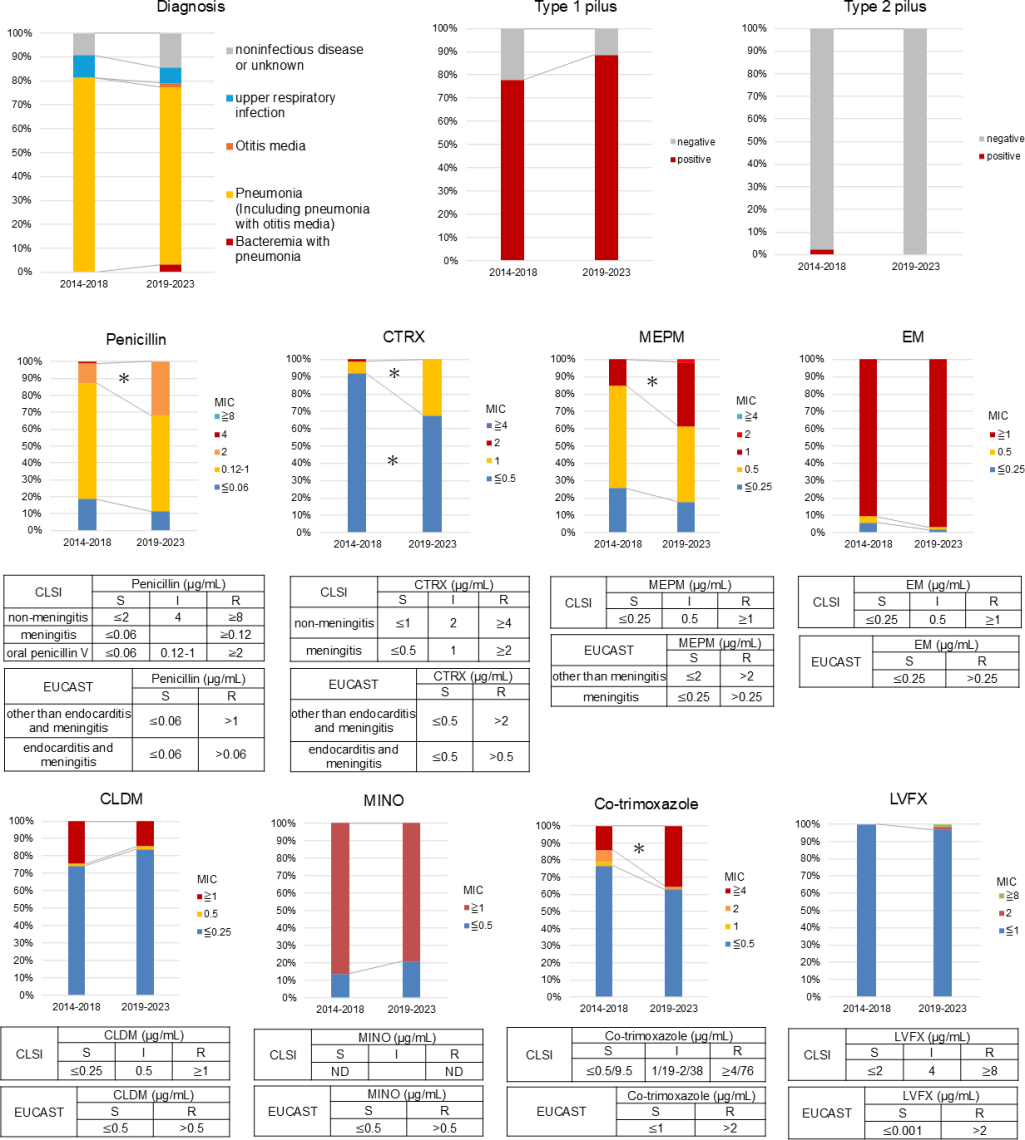
Clinical diagnosis, pilus positivity, and antimicrobial susceptibility of serotype 35B isolates from 2014 to 2018 (*n* = 86) and from 2019 to 2023 (*n* = 62). Tables show the breakpoints according to the Clinical Laboratory and Standards Institute (CLSI) and European Committee on Antimicrobial Susceptibility Testing (EUCAST). CTRX, ceftriaxone; MEPM, meropenem; EM, erythromycin; CLDM, clindamycin; MINO, minocycline; LVFX, levofloxacin; MIC, minimum inhibitory concentration; S, susceptible; I, intermediate; R, resistant. *, *P* < 0.05.

Figure 5 presents the clinical diagnosis, pilus gene positivity, and antimicrobial susceptibility profiles of all serotype 35B isolates from 2014 to 2018 (*n* = 86) and from 2019 to 2023 (*n* = 62). Most of the isolates were from cases with pneumonia (including pneumonia with otitis media). Although two strains were isolated from bacteremia in 2019 and 2020, the proportion of each diagnosis did not significantly change between the two periods (Fig. 5). Most of the 35B isolates were type 1 pilus positive and type 2 pilus negative. The differences in the positive rate of pilus between the two periods were not significant (Fig. 5). A significant increase in the proportion of isolates with a penicillin MIC of 2 µg/mL was observed (*P* = 0.002). For ceftriaxone (CTRX), the number of isolates with an MIC of 1 µg/mL significantly increased, whereas those with MICs of ≤0.5 µg/mL significantly decreased (*P* < 0.001). Similarly, the number of meropenem (MEPM) isolates with an MIC of 1 µg/mL increased significantly (*P* = 0.002). These shifts indicate a trend toward increased nonsusceptibility to β-lactams (Fig. 5). Additionally, the proportion of strains with co-trimoxazole MICs of ≥4  µg/mL increased significantly to 35.5% in 2019–2023 (*P* = 0.002) (Fig. 5). Most of the 35B isolates were resistant to erythromycin (EM) and minocycline (MINO) and susceptible to clindamycin (CLDM) and levofloxacin (LVFX), indicating their susceptibilities did not significantly change between the two periods (Fig. 5). Table 2 presents the clinical information, pilus positivity, and antimicrobial susceptibility for each ST and CC of the serotype 35B isolates. Most (14 out of 17) of the 35B-ST156 strains were isolated from children under 5 years old, and the rate was significantly higher than that of CC2755 (*P* = 0.011) (Table 2). There was no 35B-ST156 strain isolated from IPD cases, whereas 2 out of the 106 CC558 isolates were from IPD cases. The distribution of clinical diagnoses for 35B-ST156 did not differ significantly from those of CC558 or CC2755, except for a higher rate of otitis media in 35B-ST156 than that in CC558 (*P* = 0.016) (Table 2). No deaths occurred within 28 days of isolating 35B-ST156 or CC2755 (Table 2). Two cases with CC558 strains died within 28 days; however, pneumococcal disease was not the cause of their death. Among the 17 35B-ST156 isolates in this study, all exhibited penicillin MICs of ≥1 µg/mL. Notably, 64.7% had MICs of ≥2 µg/mL, significantly higher than in CC558 (18.9%) and CC2755 (0%) (*P* < 0.001). The rates of MIC ≥1 µg/mL for CTRX and MIC ≥1 µg/mL for MEPM in 35B-ST156 were significantly higher than those in CC558 and CC2755 (*P* < 0.001). All 35B-ST156 isolates were resistant to co-trimoxazole, a rate significantly higher than in CC558 (*P* < 0.001) and CC2755 (*P* = 0.006). Conversely, all 35B-ST156 isolates were susceptible to CLDM, unlike CC2755 (*P* < 0.001), and their susceptible rate to MINO was significantly higher than that of both CC558 and CC2755 (*P* < 0.001). Therefore, the change in the antimicrobial susceptibility pattern of serotype 35B isolates was primarily due to a change in the proportion of ST156 isolates (Fig. 5; Table 2).

**TABLE 2:** Clinical information, pilus positivity, and antimicrobial susceptibility for each sequence type (ST) and clonal complex (CC) of the serotype 35B isolates^*a*^*^,^*^*b*^

	ST156 *n* = 17		CC558 *n* = 106		CC2755 *n* = 22		ST156 vs CC558	ST156 vs CC2755
							*P*	*P*
Clinical information								
Age (years)								
<5	14	82.4%	68	64.2%	8	36.4%	0.139	0.011
5–64	2	11.8%	14	13.2%	6	27.3%	0.823	0.426
≥65	1	5.9%	24	22.6%	8	36.4%	0.204	0.052
Sample								
Respiratory sample	17	100%	104	98.1%	22	100%	0.644	1
Blood	0		2	1.9%	0		0.644	NA
Clinical diagnosis								
Pneumonia	14	82.4%	85	80.2%	17	77.3%	0.904	0.992
Bacteremia	0		2	1.9%	0		0.644	NA
Otitis media	3	17.6%	2	1.9%	3	13.6%	0.016	0.918
Upper respiratory infection	1	5.9%	10	9.4%	1	4.5%	0.985	0.586
Noninfectious disease or unknown	1	5.9%	11	10.4%	4	18.2%	0.889	0.512
Prognosis								
Recovery	17	100%	101	95%	22	100%	0.800	1
Died within 28 days	0		2	1.9%	0		0.644	NA
Unknown	0		3	2.8%	0		0.885	NA
Pilus positivity								
Type 1 pilus positive	17	100.0%	106	100.0%	0	0.0%	1	<0.001
Type 2 pilus positive	0		0		1	4.5%	1	0.626
Antimicrobial susceptibility								
MIC (µg/mL)								
Penicillin ≤0.06	0		1	0.9%	19	86.4%	0.293	<0.001
0.12–1	6	35.3%	85	80.2%	3	13.6%	<0.001	0.052
≥2	11	64.7%	20	18.9%	0		<0.001	<0.001
CTRX ≤0.5	2	11.8%	93	87.7%	22	100.0%	<0.001	<0.001
1	15	88.2%	12	11.3%	0		<0.001	<0.001
≥2	0		1	0.9%	0		0.293	NA
MEPM ≤0.25	0		7	6.6%	22	100.0%	0.598	<0.001
0.5	4	23.5%	75	70.8%	0		<0.001	0.029
≥1	13	76.5%	24	22.6%	0		<0.001	<0.001
EM ≤0.25	0		3	2.8%	2	9.1%	0.885	0.495
0.5	0		4	3.8%	0		0.938	NA
≥1	17	100.0%	99	93.4%	20	90.9%	0.598	0.495
CLDM ≤0.25	17	100.0%	94	88.7%	5	22.7%	0.308	<0.001
0.5	0		1	0.9%	0		0.293	NA
≥1	0		11	10.4%	17	77.3%	0.35	<0.001
MINO ≤0.5	12	70.6%	8	7.5%	3	13.6%	<0.001	<0.001
≥1	5	29.4%	98	92.5%	19	86.4%	<0.001	<0.001
Co-trimoxazol ≤0.5	0		103	97.2%	1	4.5%	<0.001	1
1–2	0		2	1.9%	7	31.8%	0.644	0.012
≥4	17	100.0%	1	0.9%	14	63.6%	<0.001	0.006
LVFX ≤2	17	100.0%	105	99.1%	22	100.0%	0.293	1
4	0		0		0		NA	NA
≥8	0		1	0.9%	0		0.293	NA

^*a*^ CC558, clonal complex 558 (ST558 and STs for which ≥5 of the 7 alleles were identical to ST558); CC2755, clonal complex 2755 (ST2755 and STs for which ≥5 of the 7 alleles were identical to ST2755); CTRX, ceftriaxone; MEPM, meropenem; EM, erythromycin; CLDM, clindamycin; MINO, minocycline; LVFX, levofloxacin; MIC, minimum inhibitory concentration; *P*, *P* value.

^*b*^ NA, not applicable.

## DISCUSSION

Pneumococcal vaccines reduce the isolation rate of vaccine-type pneumococci, whereas vaccine pressure alters the serotype distribution of the isolates. The proportion of serotype 35B has increased among clinical isolates of pneumococcal diseases in countries that have introduced vaccinations (25, 26). In this study, we collected isolates from clinical specimens regardless of the confirmed diagnosis. Since *S. pneumoniae* is a commensal bacterium, the isolates were assumed to include colonized strains. Therefore, this study investigated the spread of serotypes, either commensal or pathogenic bacteria, among populations in this region. Given that pneumococcal disease begins with nasopharyngeal carriage (27), we believe that serotype prevalence—including in asymptomatic carriers—affects their likelihood of causing disease and, ultimately, IPD. Notably, the trends in serotype distribution observed in our study closely mirrored those reported in nationwide IPD surveillance (6).

The present study showed that the major ST of serotype 35B isolates in this region recently changed to ST156. Whole-genome analysis showed that the 35B-ST156 isolates harbored type 1 pilus like ST558, the most common ST during 2014–2022, and exhibited resistance profiles for β-lactam (*pbp1a*: 4, *pbp2b*: 12, and *pbp2x*: 7), macrolide (*mefE*), and co-trimoxazole (*folA* mutation and *folP* insertion). Furthermore, the 35B-ST156 clone in this study was closely related to the 35B-ST156 strain that has spread in the United States after the introduction of PCV13 (15, 28) and has been suggested to spread within this region through additional repeated small mutations.

In our study, only 2 out of 148 35B strains were from normally sterile samples, suggesting that serotype 35B has low invasiveness despite its high isolation rate. ST156 showed no distinct clinical differences compared with other 35B sequence types, aside from being more frequently detected in children and otitis media cases, and it has not yet been isolated from sterile sites. However, in a nationwide study of the serotype distributions of IPD isolates (6), serotype 35B was the most commonly detected nonvaccine serotype in recent IPD cases. The reason for this discrepancy remains unclear. Given the small number of ST156 isolates analyzed, the clinical significance of 35B-ST156 requires further investigation.

The rising prevalence of type 1 pilus-positive serotype 35B isolates may reflect the increasing proportion of ST156, which carries the type 1 pilus gene, replacing CC2755, which lacks it. We have previously suggested that the type 1 pilus, a virulence factor for adherence, of ST558 contributes to the prevalence of serotype 35B among nonvaccine serotypes (8). Furthermore, we verified that the type 1 pilus of ST558 isolates enhanced adhesion to host cells *in vitro* (9). Therefore, ST156, which also has a type 1 pilus, may have properties that are advantageous for colonization that potently leads to pneumococcal disease.

Moreover, it is concerning that the MICs for β-lactams in 35B-ST156 isolates were higher than those of the previous serotype 35B strains. The resistance profiles for β-lactam of 35B-ST156 were identical to those of ST558 in the *pbp1a* and *pbp2x* mutations (15, 28), because 35B-ST156 strains were suggested to be caused by capsular switching between 35B-ST558 and 9V-ST156; the *pbp2x*-*cps9v*-*pbp1a* region of 9V-ST156 was replaced with the corresponding sequences from 35B-ST558 through recombination events. The 35B-ST156 isolates had *pbp2b* mutations different from those of ST558. Li et al. reported correlations between β-lactam MICs and PBP types in *S. pneumoniae* (29). Therefore, *pbp2b*:12 in 35B-ST156 may contribute to a higher β-lactam resistance rate than that in ST558, thereby contributing to survival in hosts. In contrast, CC2755, a unique β-lactam–susceptible clone found in Japan and neighboring countries, has decreased in prevalence.

Additionally, an increased proportion of 35B-ST156 altered the antimicrobial susceptibility pattern of serotype 35B isolates. Like CC558 and CC2755, 35B-ST156 exhibited high resistance rates to EM. However, it showed significantly greater resistance to co-trimoxazole than either clone, contributing to its multidrug-resistant phenotype. Conversely, 35B-ST156 remained highly susceptible to CLDM, unlike CC2755, and to MINO, unlike both ST558 and ST2755 (Table 2). If the proportion of ST156 continues to increase, resistance rates of serotype 35B to β-lactams and co-trimoxazole may increase further, while resistance to CLDM and MINO may decrease. These trends underscore the need for ongoing surveillance of 35B-ST156.

Despite our insightful findings, this study had several limitations. First, the study was conducted at a single sentinel hospital; therefore, geographical variations cannot be accounted for, and the findings may not be representative of a nationwide trend. However, given the characteristics of 35B-ST156 and its spread in the United States after the introduction of PCV13, similar changes are expected to have occurred in Japan. Second, because isolates were collected regardless of clinical diagnosis, many may represent colonizing rather than disease-causing strains. However, the spread of colonizing *S. pneumoniae* remains clinically relevant, as it can precede and contribute to disease, particularly in older adults and individuals with risk factors. In the national IPD surveillance of adults in Japan, serotype 35B accounted for a small percentage of IPD isolates in 2014; however, its proportion has increased annually, and it became the most common serotype among nonvaccine serotypes by 2022 (6). Notably, 35B-ST156 was not detected in that surveillance until 2017 (30), and only 1 ST156 isolate was identified among 106 serotype 35B IPD isolates in another Japanese IPD surveillance study from 2010 to 2017 (31). Although our findings reflect a limited geographical region, they suggest a growing prevalence of 35B-ST156 in Japan. PCV15 and PCV20 were introduced in Japan in 2023 and 2024, respectively; however, serotype 35B was not covered by these vaccines.

In conclusion, the distribution of 35B-ST156 among *S. pneumoniae* serotype 35B isolates has increased in a region of Japan and has altered the antimicrobial resistance pattern of serotype 35B. This indicates that serotype 35B-ST156, which is piliated and multidrug resistant, may have spread nationwide and that the distribution of STs in nonvaccine serotypes may alter the antimicrobial susceptibility of *S. pneumoniae* in Japan. Our study provides useful information for guiding the treatment and prevention of pneumococcal infections. Continued nationwide monitoring of pneumococcal sequence types is essential, as ST distribution directly influences bacterial behavior and antimicrobial resistance profiles.

## Data Availability

Sequence data generated during this study were deposited in GenBank under accession numbers JBDQYR000000000–JBDQYU000000000 and in DDBJ under accession numbers DRR628322–DRR628334.
